# Effectiveness of UEFA's regulation for European football financial management: A comprehensive systematic review and meta-analysis

**DOI:** 10.1016/j.heliyon.2024.e39151

**Published:** 2024-10-09

**Authors:** Jorge Martín-Magdalena, Susana De los Ríos-Sastre, Raquel Redondo, David Alaminos

**Affiliations:** a Universidad Pontificia Comillas Alberto Aguilera 23, 28015, Madrid, Spain; b University of Barcelona, Avda. Diagonal, 690, 08034, Barcelona, Spain

**Keywords:** Financial fair play, Financial performance, Football, Systematic review, Meta-analysis

## Abstract

**Background:**

The Union of European Football Association's (UEFA) Financial Fair Play (FFP) regulations are a topic of ongoing debate. This study aims to evaluate and quantify the FFP's effect on clubs' financial performance and critically review the regulatory changes introduced by the UEFA in 2022 through the new Financial Sustainability Regulations (FSR).

**Methods:**

A systematic review and meta-analysis were conducted using the Web of Science and Scopus databases up to December 31, 2023, following the Preferred Reporting Items for Systematic Reviews and Meta-Analysis (PRISMA) statement.

**Results:**

This systematic review included 22 articles; a meta-analysis was conducted on 52 financial measures derived from 20 studies of 11 articles. The meta-analysis’ main result—obtained by comparing two different financial measures (profitability versus solvency)—revealed that FFP's effect on profitability measures was significant, with a value of 0.151 (*p* = 0.050); however, the effect size for solvency measures was not significant, with a value of 0.049 (*p* = 0.639).

**Contributions:**

This systematic review revealed variability in the results of the studies analysed, reflecting contextual factors' influence, which underlines the need for a more adaptive and specific approach to clubs’ financial control policies. The meta-analysis found that the type of financial measure employed (profitability versus solvency) was a notable source of variability among the studies, as its moderating effect was significant. Consequently, the FFP exerted contrasting effects on profitability and solvency.

**Limitations:**

A significant level of heterogeneity was observed in the financial measures analysed, predominantly because of the different samples and periods across the included studies.

**Conclusions:**

This study corroborates FFP's mixed and limited impact on financial performance, highlighting the need for stricter control in European football, which aligns with the new FSR. Our study underscores aspects that future research should address to deepen knowledge of UEFA regulations' efficiency in enhancing football's financial sustainability.

## 1 Introduction

The European football industry operates within a highly competitive promotion-relegation system, requiring significant club investments in talented players [[1], [2], [3], [4]]. Unlike traditional profit-maximising companies, European football's economic model has historically prioritised sporting success over financial performance, precipitating significant financial challenges that triggered regulatory intervention by the Union of European Football Association (UEFA).

These issues include overspending and debt accumulation, with numerous clubs pursuing short-term success and, spending more than they earn, leaving them in precarious financial positions. Moreover, inflation in the transfer market, driven by competition for the best players, has contributed to unsustainable financial practices. Several clubs rely on external funding, which results in long-term instability if this support is withdrawn or if the results fail to fulfil expectations. Inconsistent financial management, characterised by poor governance and weak revenue management, has contributed to the financial distress of numerous clubs that prioritise immediate success over long-term sustainability [1,2,[5], [6], [7], [8], [9], [10], [11]].

Furthermore, marked economic disparities prevail between clubs, with larger clubs enjoying greater revenues from television rights and sponsorships, exacerbating financial inequality with small clubs as they encounter higher economic instability owing to limited market access [2,8,10,[12], [13], [14], [15]]. Academic literature has highlighted the importance of football clubs’ prudent financial management for long-term financial sustainability [7,16] and the need for effective regulation for economic control in the football industry.

Responding to these financial challenges, the UEFA approved the *UEFA Club Licensing and Financial Fair Play Regulations* in 2009 [17], known as the Financial Fair Play (FFP) regulation. Notably, FFP was introduced in the context of growing concern regarding financial sustainability in European football at the European level. Although national leagues had already begun implementing measures, FFP was important because it represented a unified European effort to ensure that clubs participate in European competitions within a sustainable financial framework, being the first economic control regulation to be applied in a uniform and coordinated manner at the European level by the UEFA. The FFP's main objectives are promoting European football clubs' long-term financial stability, reducing financial difficulties' risk, and ensuring that clubs operate within their means on the same level playing field [9,10,14,17].

The FFP included the following two main requirements: (i) the ‘break-even’ rule limited clubs to losses of no more than €5 million over three years, extendable to €30 million if owners/shareholders covered the additional losses, ensuring that football clubs do not spend more than their income; (ii) the ‘no-overdue-debt' rule required clubs to pay debts on time and maintain manageable debt levels. Therefore, the FFP requires clubs to balance their financial accounts by imposing restrictions on excessive spending and debt accumulation and, encouraging more responsible financial management, which helps reduce their financial insolvency risk, crucial to ensuring the long-term financial sustainability of clubs and the European football ecosystem.

Since their introduction, compliance with the UEFA's financial regulation has become an essential challenge for European football clubs' management [16,[18], [19], [20], [21]] because failure to comply with the rules after an annual evaluation process resulted in penalties for clubs, such as financial fines or exclusion from competitions.

Despite its well-intended purpose, its adequacy has received criticism owing to its implementation, and considerable debate has persisted regarding its positive impact on football clubs' economic and financial performance. On the one hand, FFP's efficacy and legality have been questioned from a regulatory perspective, even as criticising the UEFA's overly prominent role as a governance body for the European football industry [22]. On the other hand, the academic literature [9,[23], [24], [25], [26], [27], [28], [29]] provides evidence of the FFP's unclear impact on football clubs' financial sustainability.

**Fig. 1:**
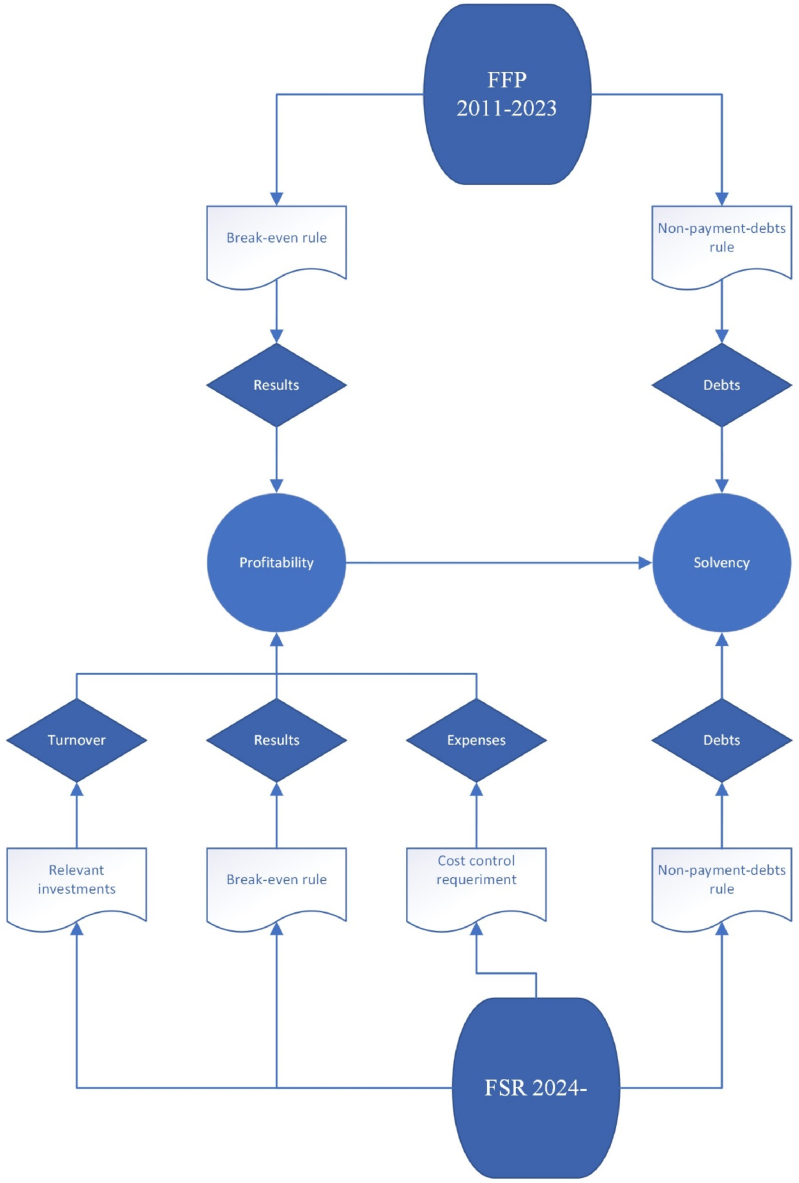
UEFA economic control regulations FSR vs FFP.

Within this framework, the UEFA introduced *UEFA Club Licensing and Financial Sustainability Regulations* (FSR) in 2022 [30], replacing the previous FFP [17,31]. The new FSR maintains the aim of long-term financial sustainability for European football clubs, which involves enhancing discipline and transparency and enforcing monitoring requirements (Fig. 1). Specifically, the ‘break-even’ rule and the requirements to control debt are maintained, though the new regulation allows clubs to incur losses up to €60 million over a three-year interval extendable to €90 million if the club demonstrates significant investments in infrastructure, youth development, or women's football. Additionally, the new regulation includes a new key element of ‘cost control’, limiting spending on players and coach salaries, transfers, and agent fees to 70 % of a club's revenue and stricter enforcement of timely payment obligations.

A decade after implementing the FFP rules, a lack of consensus prevails regarding the UEFA financial regulation's effects on European football clubs' financial performance. The findings in the current scholarly literature on this topic are inconclusive and occasionally contradictory, depending on the league, football club, or period under investigation. Empirical evidence seems to spark controversy in discussions regarding UEFA regulations [9,27,28] and determining the FFP's overall impact on financial performance has proven challenging because of variability in the methodologies, theories, samples and metrics employed in previous studies. Therefore, understanding the prevailing scenario in this regard is necessary to make decisions that help improve European football's financial sustainability.

Owing to the absence of a thorough evaluation and variability in existing empirical studies on UEFA regulations' effectiveness, we aim to bridge this gap by conducting a comprehensive systematic review and meta-analysis of published studies to examine the FFP regulation's impact on European football clubs' financial performance, attempting to identify variables that may explain the lack of research consensus and the substantial variability among studies. We propose that one of these variables is the type of measure used (profitability versus solvency). Profitability and solvency are commonly used measures for evaluating financial performance [32,33] and are directly linked to two main aspects of FFP—namely, the break-even and no-overdue-debt rules. We believe that analysing these measures separately is valuable as the FFP's impact on them may differ. Clubs may prioritise their short-term financial results (profitability) over their medium- and long-term financial stability (solvency), precipitating varying impacts.

Our study demonstrates that the UEFA's FFP regulation has exerted mixed and limited effects on European football clubs' financial performance. Moreover, the meta-analysis reveals an important insight that these regulations exert contrasting effects on club profitability and solvency. Specifically, a significant positive impact on profitability is observed but not a significant impact on solvency, suggesting that adhering to the break-even requirement positively affects football clubs' profitability. However, the profitability improvement has not been sufficiently consistent to enhance solvency, especially when combined with insufficient progress vis-à-vis the non-overdue debt rule. These results imply that clubs primarily achieve the break-even point by increasing profits through player transfers, not through relatively effective financial management. The FFP regulation has marked significant advancements in the football industry's economic governance; however, its capability to uphold financial stability within the European football industry remains limited. Nevertheless, we believe that the stringent requirements outlined in the new FSR regulation, particularly the cost control rule, present a more optimistic outlook.

This study contributes to the current literature by providing a comprehensive overview of the global effect of the UEFA's regulations on financial performance and offering new variables that explain the high variability among studies. To our knowledge, this is the first study to synthesise existing empirical evidence on this specific topic and aggregate relevant data from multiple studies through a meta-analysis. This study helps identify limitations in the existing literature to guide future research and provide recommendations for improving economic control policies in European football.

## 2 Methodology

We conducted a comprehensive systematic review and meta-analysis of published studies following the Preferred Reporting Items for Systematic Reviews and Meta-Analyses (PRISMA) guidelines to ensure the transparency and reproducibility of the process [34,35] (see Appendix A for the PRISMA Checklist). A systematic review and meta-analytical techniques [36,37] were applied to synthesise and evaluate existing empirical studies on the FFP regulation's impact on European football clubs' financial performance and integrate and quantify this effect on profitability and solvency.

### 2.1 Literature search and selection criteria

A search strategy was developed following prior reviews in related fields [[38], [39], [40]] to identify appropriate studies for inclusion. We focused on indexed publications for their scientific reliability [41], aiming to gather all significant literature on how the FFP regulation has influenced European football clubs' financial performance. Previously, we analysed studies on FFP (e.g. Refs. [10,20,42]) and football clubs' financial performance [2,7] to identify relevant keywords. We compared the results of using three different word sequence terms for the search—specifically, (a) ‘(Financial OR economic OR business OR performance OR ∗solvency OR ∗debt∗ OR profit∗ OR ratio OR indicator OR sustainability OR control) AND (football OR soccer OR club OR UEFA OR league OR professional OR team∗)’; (b) (‘Financial performance’ OR ‘economic performance’ OR ‘business performance’ OR performance OR ∗solvency OR ∗debt∗ OR profit∗ OR ratio OR indicator OR ‘financial sustainability’ OR ‘economic sustainability’ OR ‘business sustainability’ OR ‘economic control’ OR ‘financial control’) AND (football OR soccer OR club OR UEFA OR league OR professional OR team∗); and (c) ‘(Financial OR economic OR business) AND (performance OR ∗solvency OR ∗debt∗ OR profit∗ OR ratio OR indicator OR sustainability) AND (football OR soccer OR club OR UEFA OR league OR ‘professional football’ OR team∗) AND (‘Fair play’ OR ‘economic control’ OR ‘financial control’)’. Thereafter, we decided to only include the term ‘Financial Fair Play’ in the search field because the outcomes were the most adjusted to our study's aim.

A search was conducted to obtain titles, abstracts, and keywords from the Social Sciences Citation Index (1956–present) within the Web of Science (WoS) and Scopus databases. Both WoS and Scopus are high-quality databases with clear advantages; WoS stands out because of its rigorous selection criteria for publication inclusion, which is typically considered a guarantee of data reliability and relevance, whereas Scopus is valued for its coverage and detailed citation analysis [43]. Therefore, they are complementary databases, particularly for comprehensive literature reviews and multidisciplinary research [44].

No language or time restrictions were imposed. We recovered all searches up to December 31, 2023 and identified 175 articles for possible inclusion, from which we eliminated duplicates in both databases (n = 55). Additionally, we manually performed cross-citation analysis to verify these articles' reference lists and detect additional eligible studies. Following this analysis, 3 new articles were included. In total, 123 articles were retrieved. Three authors (JMM, SDLR and DA) independently reviewed the selected articles' titles and abstracts according to the following inclusion criteria: 1) published in peer-reviewed journals; 2) examining the impact of the UEFA's FFP regulation on professional football clubs' financial performance, excluding studies explicitly investigating its effects on clubs' competitive performance; and, finally, 3) only empirical papers, both qualitative and quantitative. Any disagreement in the selection process was resolved through discussion with the fourth author (RR). This screening—based on the aforementioned inclusion criteria—resulted in 101 articles being excluded and 22 articles being considered eligible for further analysis.

**Fig. 2:**
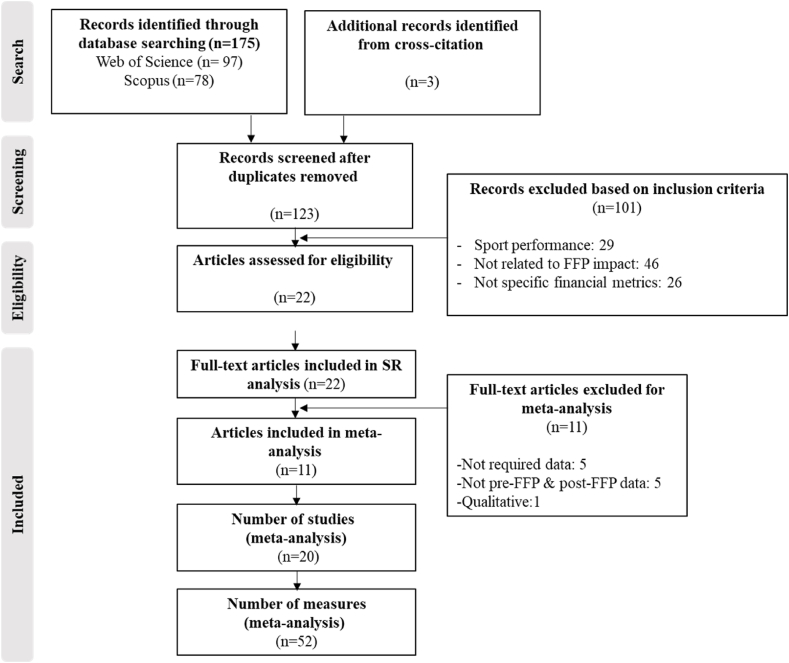
PRISMA flow diagram.

The authors independently examined these 22 articles' full texts and agreed to include them in the systematic review. Subsequently, each article's full text was reviewed again and individually coded by three authors (JMM, SDLR and DA), and the data were entered into Excel files. Finally, two additional inclusion criteria for the meta-analysis were considered: 1) only papers with quantitative analysis and 2) papers with data on financial measures of financial performance (profitability and solvency) before and after FFP implementation. Consequently, based on these criteria, the meta-analysis included 11 articles. Fig. 2 illustrates a flow diagram of the study selection process.

### 2.2 Coding process

Data were extracted using a standardised coding form as an information-gathering instrument [45]. For the systematic review, the 22 articles were coded for 14 predefined fields—specifically, author, journal, year of publication, number of studies, objectives, hypothesis, country, league, seasons covered, sample, methodology, applied metrics, results, and key findings. Thereafter, the results were segmented into two financial categories—namely, profitability and solvency. Further coding was conducted for the 11 articles included in the meta-analysis to extract the following data for the profitability and solvency measures and pre-FFP and post-FFP periods: means, standard deviations, sample sizes and number of years analysed. Additionally, we computed the temporal distance between the median years of the pre-FFP and post-FFP periods and coded whether the study was conducted in a single country versus several countries. Three authors (JMM, SDLR and DA) independently conducted this coding process, and any doubts or disagreements in the process were resolved through discussion with the fourth author (RR). During this process, we identified two studies with missing standard deviations ([27]; England and France), for which we followed the prognostic method outlined by Ma et al. [46] to estimate the missing standard errors of the mean, enabling us to include as many measures in the analysis as possible. This estimation allowed us to analyse 11 articles, 20 studies (samples) and 52 measures.

### 2.3 Meta-analysis strategy

We determined the effect sizes for all selected studies according to the aforementioned inclusion criteria using Cohen's *d* as the standardised mean difference, calculated as the difference between the means for the financial measures in the pre-FFP and post-FFP periods divided by the pooled standard deviation.

In this regard, to incorporate the different measures of the effect of the FFP regulation extracted, even if they originated from a single sample, we used a three-level meta-analysis model, as this model can deal with dependent effect sizes within studies [47,48]. In this approach, the following three different variation levels are considered: level one accounts for the sampling variation for each effect size; level two encompasses the variation over outcomes/measures/effect sizes within a study (we equated a study to all the analyses originating from the same sample); level three accounts for the variation over studies (different samples). The corresponding model's specification is as follows:

[1] $$d_{jk}=\beta_{jk}+r_{jk}\;where\;r_{jk}∼N\left(0,\sigma_{r_{jk}}^{2}\right)$$

[2] $$\beta_{jk}=\theta_{0k}+\nu_{jk}\;where\;\nu_{jk}∼N\left(0,\sigma_{\nu }^{2}\right)$$

[3] $$\theta_{0k}=\gamma_{00}+u_{0k}\;where\;u_{0k}∼N\left(0,\sigma_{u}^{2}\right)$$

In the first equation (at level one, the sample level for each effect size), $d_{jk}$ is the j^th^ observed effect size (*j* = 1, 2, …, *J*) from study *k* (*k* = 1, 2, …, *K*). This equation indicates that observed effect sizes $d_{jk}$ are spread around the underlying population effect size $\beta_{jk}$, with sampling error $r_{jk}\text{.}$ These errors supposedly follow a normal distribution with zero mean and variance $\sigma_{r_{jk}}^{2}$ (depending on the study and its outcome). The second equation is at level two or the level of the outcomes/measures. It indicates that the population effect for the different outcomes within a study $\beta_{jk}$ is the sum of the study mean ($\theta_{0k}$) and residuals ($\nu_{jk}$). The third equation is at level three or the study level (different samples). It indicates that the study mean effects are decomposed in terms of an overall mean ($\gamma_{00}$) plus the corresponding residuals.

Meta-analysis was conducted using R (R Core Team, 2017) and the R packages metafor [49] and metaforest, following Assink and Wibbelink's [50] steps and recommendations. Thus, we determined the overall effect size (reported as Cohen's *d*) with a 95 % CI, heterogeneity (reported using the Q-test and *p* values), variance decomposition and significance of the variance components. As multilevel model parameters are typically estimated and tested using maximum likelihood estimation procedures, we selected the restricted maximum likelihood procedure because this method decreases the bias in the variance component estimates [48,50]. Thereafter, to investigate effect size heterogeneity and its possible causes more deeply, a second set of moderation analyses was performed to offer a wider perspective on the FFP regulation's effect.

## 3 Results

### 3.1 Overview of included studies

Table 1 presents a synopsis of the main characteristics of the 22 articles included in this systematic review, of which only 11 matched our inclusion criteria for the meta-analysis. These studies used various methods, theories, sample sizes, data sources, and statistical techniques to examine financial performance. These papers were published in 15 journals (Appendix B). Since the FFP regulation's implementation in 2011, scholarly interest in this area has progressed considerably, with 69 % of the articles published in the last five years.

**Table 1:** Overview of included studies.

	Paper	Theory	League/Club	Seasons	Data	nº clubs	Statistical technique	N	Dependent variable	Cat. of measures	FFP	Key findings
1	Acero et al. (2017)	Agency	Big Five leagues	2007–2013	Panel data from financial accounts	94	ANOVA and panel regression analysis	562	ROA and ROS	P	FFP by temporal dummy	FFP regulation can initially act as a control mechanism for improving clubs' profitability, but it does not effectively address the problem of minority shareholders since high ownership concentration negatively affects financial performance.

2∗	Ahtiainen & Jarva (2022)	Agency, Profit Maximization, Utility Maximization and Soft Budget Constraint	Big Five leagues	2008–2016	Panel data from financial accounts	139	Regression analysis: Logit and OLS models	1094	Probability of reporting a loss for a football club, PBT or EBIT	P and S	FFP dummy variable	After introducing the FFP, the probability of a club incurring financial losses in the analysed leagues has decreased. The Spanish league also has a positive and significant effect on increasing the clubs' profitability.

3∗	Barajas et al. (2017)	Agency, Profit Maximization, and Utility Maximization	Biggest European football clubs by revenues	2011, 2012, 2013	Panel data from financial accounts	10	Multicriteria Decision Analysis (MCDA)	30	Financial stability and sustainability ratios	P and S	Three periods, pre-FFP (2011) and post-FFP (2012 and 2013)	After introducing the FFP, the financial ratios of profitability, solvency, coverage, and liquidity for the top ten clubs (on average) improved from 2011 to 2013.

4	Dermit-Richard et al. (2019)	Agency and Soft Budget Constraints	French Ligue1	2005–2014	Panel data from financial accounts	24	Club individual accounts descriptive analysis	139	Net profit and other financial indicators	P	France DNCG regulations vs UEFA FFP	French clubs are subject to the FFP and the French league's regulation (DNCG). While the FFP focuses more on sustainability and profitability, the DNCG focuses on solvency, allowing clubs to cover their long-term investments (such as player transfers) with shareholder contributions. The results show that compliance with the DNCG rules is not helping French clubs achieve FFP compliance and improve their financial performance.

5∗	Dimitropoulos & Koronios (2018)	Agency	Belgium, Finland, France, Greece, Italy, Netherlands, Norway, Spain, and UK	2008–2016	Panel data from financial accounts	109	Panel regression analysis	844	ROA	P	FFP dummy variable and two sub-periods: pre-FFP and post-FFP	After introducing the FFP, the clubs' profitability in the analysed leagues has not improved, observing a decrease in the persistence of profits. However, the predictability of profits has increased.

6∗	Dimitropoulos & Scafarto (2021)	Agency and Soft Budget Constraints	Italian SerieA	2007–2017	Panel data from financial accounts	15	SUR Regression analysis	165	Financial performance (revenues, operation profit and net profit)	P and S	FFP dummy variable and two sub-periods: pre-FFP and post-FFP	Introducing the FFP positively and significantly affects the Italian clubs' financial performance through the profits from selling player rights.

7∗	Fernández-Villarino & Domínguez-Gómez (2022)	Agency	Spanish LaLiga	2011, 2015	Panel data from financial accounts	44	A quasi-experimental study	88	Financial performance by financial indicators	P	Two sub-periods, pre-FFP and post-FFP	Introducing the FFP significantly and positively affects Spanish clubs' financial profitability performance.

8	Franck (2018)	Soft Budget Constraint	Clubs in the UEFA Champions League	2012–2017	Data from financial accounts	–	Descriptive Analysis of the financial situation post-FFP	–	Financial indicators of FFP rules: revenue growth, overdue payable, operating profits, net results and net equity	P	Two sub-periods, pre-FFP and post-FFP	Following the introduction of the FFP, the largest clubs have reported positive operating profits and increased their net equity (assets minus liabilities), increasing the economic gap between the top clubs and the rest. However, the results suggest a polarization process between the largest clubs and the rest.

9∗	Francois et al. (2022)	Agency, Profit Maximization, Utility Maximization and Soft Budget Constraint	English Premier League and French Ligue 1	2008–2018	Panel data from financial accounts	72	Financial indicators descriptive analysis	395	Profitability and cost efficiency indicators	P	Two sub-periods, pre-FFP and post-FFP	After introducing the FFP, there has been a positive and significant increase in the break-even point and profitability of EPL clubs (Europe-oriented and non-European-oriented) and non-European-oriented clubs of the French Ligue1.

10∗	García-del-Barrio & Agnese (2023)	–	English Premier, Spanish, Italian and French leagues	2010–2019	Panel data from financial accounts	40	Panel regression model	796	Wages/Revenues ratio	P	Seasons pre-FFP and post-FFP	The FFP regulation helps mitigate the financial risk of clubs and encourages a change in the management mentality of managers that also positively impacts better sports management.

11	Ghio et al. (2019)	X-inefficiency	Italian Serie A	2005–2015	Panel data from financial accounts	33	SFA and DEA regression analysis	330	Total operational cost	P	Two subperiods: pre-FFP and post-FFP	After introducing the FFP, there have been no significant effects on Italian clubs' profitability, although the cost-efficiency gap between the clubs has decreased.

12	Jakar & Gerretsen (2021)	Agency, Profit Maximization and Utility Maximization	Clubs in the UEFA Champions League	2006–2008	Panel data from financial accounts	–	Time series and ordered logistic regression model	910	Prize money in the UEFA Champions League	P	Two subperiods: pre-FFP and post-FFP	Implementing Financial Fair Play can increase the gap between the largest clubs and the rest because the largest have more chances and capacities to succeed in sports and increase their turnover.

13	Maclean et al. (2022)	Resource Dependency Theory and Private Interest theory of regulation	Scottish League	2018	In-depth semi-structured interviews	–	Qualitative	–	–	P	–	The introduction of the FFP has had a different impact on the larger clubs than on the rest. Large clubs compete in several overlapping competitions and have more capacity to adapt to the regulations. So, the sporting and economic distance between the biggest and the rest will increase.

14∗	Martín-Magdalena et al. (2023)	Profit Maximization and Utility Maximization	Spanish LaLiga	2008–2019	Panel data from financial accounts	22	Panel regression model	203	Profitability, Liquidity and Solvency measures	P and S	FFP dummy variable	The introduction of the FFP has increased the economic inequality between large and small clubs. Only small clubs increased their profitability, and only medium-sized clubs improved their solvency.

15∗	Neri et al. (2021)	Agency and Earnings management	Italian Serie A	2005–2018	Panel data from financial accounts	38	Panel regression model	275	Net capital from player transfers weighted by total assets	P and S	FFP dummy variable	After introducing the FFP, the profitability of the Italian clubs decreased, and their indebtedness increased.

16	Nicoliello & Zampatti (2016)	Profit Maximization and Utility Maximization	Italian Serie A	2011–2013	Panel data from financial accounts	15	Panel regression model	45	Profitability by net earnings	P	FFP dummy variable	Implementing the FFP had a positive and significant effect on the profitability of Italian clubs since it had a negative and significant impact on reducing staff costs and the rest of expenses over turnover.

17	Özaydin (2020)	–	Russian league	2008–2019	Panel data from players' transfer operations	–	Discontinuity regression analysis	2083	Players' Transfer Results		FFP dummy variable	The main impact of implementing FFP is that the break-even point is significantly effective and negative on player transfer spending and significant and positive on player transfer balance in the Russian league.

18	Peeters & Szymanski (2012)	Vertical Restraints, Profit Maximization, Utility Maximization and Soft Budget Constraint	English football	1994–2010	Panel data from financial accounting	87	Panel regression model	1010	Ratio salary cost-turnover	P	Pre-FFP and estimation of post-FFP	After the approval of the FFP, the salary turnover ratio would have fallen by up to 15 %.

19	Peeters & Szymanski (2014)	Agency, Vertical Restraints, Profit Maximization and Utility Maximization	English Premier League, Spanish LaLiga, Italian SerieA and French Ligue1	1998–2011	Panel data from financial accounts	–	Panel regression model	23,592	Salary cost	P	Pre-FFP and estimation of post-FFP	The FFP break-even rule may favor large European clubs, which access large local markets. Suppose smaller teams are not allowed to invest in improving their sporting performance through operating losses. In that case, it is difficult for them to improve their competitive performance with the big clubs.

20∗	Plumley et al. (2021)	–	English Premier League & English Football League Championship	2002–2019	Panel data from financial accounts	43	Descriptive analysis	1622	Z-Altman	P and S	Two subperiods: pre-FFP and post-FFP	Despite introducing the FFP, the results show that English clubs perform poorly financially, with a high risk of financial vulnerability, which is higher in the English second division compared to the English Premier League.

21	Urdaneta et al. (2021)	Agency and Utility maximization	Spanish LaLiga	2015, 2016, 2019	Panel data from financial accounts	28	Panel regression model	84	Transparency index	P and S	Post-FFP	After implementing the FFP, the Spanish LaLiga clubs have improved their financial performance through better profitability, solvency, and financial leverage.

22∗	Urdaneta et al. (2023)	Agency and Utility maximization	Spanish LaLiga	2014, 2017, 2020	Panel data from financial accounts	25	Panel regression model	75	Profitability, Liquidity and Solvency measures	P and S	Two subperiods: pre-FFP and post-FFP	After implementing the FFP, the Spanish LaLiga clubs have improved their financial performance through better profitability and solvency

Notes: ∗ Indicate studies included in the meta-analysis. Category of measures: Profitability (P) and solvency (S). N = number of observations.

Regarding financial performance, we identified a predominant focus in the profitability analysis of most reviewed articles. However, solvency analysis was relatively scarce, observed in only eight articles [[22], [23], [24], [25],[51], [52], [53], [54]]. Concerning the methods, most studies employed panel data based on financial accounts (20 articles), as expected considering the topic studied herein. Of these, 15 articles applied regression analysis based on panel data regression models (with different variations, including panel regression, with fixed and random effects; pooled regression and logistic regression [55]) and four articles used descriptive analysis based on data from financial accounts [56]. Only Barajas et al. [51] applied a different model based on multi-criteria decision analysis. Finally, only one article used a qualitative methodology through in-depth semi-structured interviews [57]. The number of clubs included and observations in the samples varied substantially among the papers. For example, Plumley et al. [53] included 43 clubs and 1622 observations, whereas Nicoliello and Zampatti [3] included 15 clubs and 45 observations. Finally, seven articles used FFP as a dummy variable [22], and nine segmented their data into pre- and post-FFP subperiods to evaluate its impact [14].

Agency theory was the most frequently addressed theory—noted in 13 papers. Additionally, the results indicate that the theories of utility maximization, profit maximization, and soft budget constraint appeared in 10, 8, and 7 papers, respectively. Additionally, two articles were based on the theory of vertical restrictions. Finally, three articles were based on the theories of X-inefficiency, resource dependency, private interest of regulation, and earnings management.

Our comprehensive systematic review organised and classified the main findings in the papers analysed into the following four main dimensions: (i) FFP's impact on profitability and solvency, (ii) impact on the clubs' financial management, (iii) variable effects on financial measures by country and league, and (iv) economic inequality between large and small clubs.

Regarding FFP's impact on profitability and solvency, several studies indicated that the FFP's introduction has positively impacted clubs' profitability and financial stability. For example, Acero et al. [55], for the 2007–2013 period, and Ahtiainen and Jarva [23], for the 2008–2016 period, observed improvements in clubs' profitability (measured by ROA, ROS, PBT, or EBIT) in the Big Five Leagues after the FFP's implementation, especially in leagues such as the Spanish league; FFP significantly and positively affected the profit before tax margin in Ahtiainen and Jarva's study [23].

Moreover, studies such as that by Barajas et al. [51] for the transitory period 2011–2013 observed an improvement in the financial performance of the largest clubs —such as Real Madrid, FC Barcelona, Bayern Munich, Manchester United, Manchester City, Arsenal, Juventus, AC Milan, Borussia Dortmund, and Liverpool—as measured through profit, stability coverage, liquidity, and spending ratios. By contrast, Dimitropoulos and Koronios [58] highlighted that after introducing the FFP for the analysed period —specifically, 2008–2016—to clubs of several leagues such as Belgium, Finland, France, Greece, Italy, the Netherlands, Norway, Spain, and the UK, the club's profitability (measured by ROA) exhibited no improvement, instead observing a decrease in the persistence of profits. Dermit-Richard et al. [56] observed similar results for clubs of the French league for the 2005–2014 period, suggesting that the FFP's effect may not be uniformly positive across all leagues and clubs.

Concerning the impact on the clubs’ financial management, authors such as García-del-Barrio and Agnese [59] for the 2010–2019 period for English, Spanish, Italian, and French clubs; and Peeters and Szymanski [60] and for the 1994–2010 period for English clubs, suggested that the FFP has encouraged a change in the management mentality within the clubs, promoting more prudent and sustainable management predominantly by reducing salary costs over turnover.

Furthermore, this idea was supported by Urdaneta et al. [54,61], who found that the FFP's implementation improved the clubs' transparency (measured by a transparency index) and financial management (measured by profitability, liquidity and solvency ratios), particularly in the Spanish LaLiga for the 2014–2020 period. Likewise, Nicoliello and Zampatti [3]—examining Italian clubs for the transition period 2011–2013—found that the FFP's implementation significantly and positively affected Italian clubs' profitability, predominantly by reducing salary costs over turnover. However, studies such as that Dimitropoulos and Scafarto [52], on Italian clubs for the 2007–2017 period and Özaydin [62], on the Russian league, suggested that the improvement in financial performance after the FFP's implementation has been through incentivising clubs to boost their ‘relevant income’ by increasing the gains of player transfer rights and, thereby, comply with the break-even rule.

Regarding the variable effects on financial measures by country and league, the FFP's impact seems to vary significantly depending on the country and league examined. In leagues such as the Italian Serie A and Spanish LaLiga, some studies observed significant improvements in financial performance [52,63], while others, such that by Neri et al. [26], observed that after the FFP's introduction, the Italian clubs' profitability decreased, and while their debt increased. The results are more inconsistent in other leagues, such as the study examining the English Premier League and French Ligue 1 from 2008 to 2018, which found a positive effect on English clubs' profitability but only in non-European-oriented clubs of the French League [27]. Likewise, Plumley et al. [53] highlighted that despite introducing the FFP, English clubs exhibit poor financial performance with a high risk of financial vulnerability, especially in the English second division. These results indicate that FFP's impact is not the same across all leagues.

Concerning economic inequality between clubs, concern is growing regarding economic and sporting polarization between the largest clubs and the rest, exacerbated by the FFP. In this regard, Franck [14], for clubs that participated in the UEFA Champions League from 2012 to 2017, and Jakar and Gerretsen [64], for the same clubs but during the 2006–2008 period, that is, before the FFP's introduction, highlighted that FFP may increase the gap between the largest clubs and their competitors, limiting the latter's ability to compete on a sporting and economic level. Likewise, studies such as that by Martín-Magdalena et al. [9] on Spanish clubs for the 2008–2019 period highlighted that the FFP increased economic inequality between large and small clubs (analysed by the Gini index), even though profitability and solvency improvements are observed in smaller and medium-sized clubs, respectively. However, although Ghio et al. [65] did not find significant effects of FFP on Italian clubs' profitability during the 2005–2015 period, they noted a decrease in the cost-efficiency gap between clubs, indicating that the FFP's impact on economic inequality may vary depending on each league's specific context.

In summary, our systematic review indicates that implementing the UEFA's regulations has yielded mixed effects on football clubs' financial performance. These effects vary depending on the country, club type, and period analysed.

### 3.2 Meta-analysis results

To aggregate the systematic review's results and quantitatively evaluate the FFP regulation's effect on European football clubs' profitability and solvency, a meta-analysis was conducted based on data extracted from 20 studies. Overall, 52 financial measures were obtained, with 32 focusing on profitability and 20 on solvency. Of these, 46 were from single countries, while 6 were from multiple countries (Table 2).

**Table 2:** Expanded data on meta-analysis studies and measures.

Paper	Country/League	Financial measure	Category of measure	Years pre-FPP analysed	Years post-FPP analysed	Average temporal pre-post distance	Nº clubs (pre FFP)	Nº clubs (post FFP)	Effect size
Ahtiainen & Jarva (2022)	Germany (Bundesliga)	EBIT margin	Profitability	4	5	5	37	43	0.165
Ahtiainen & Jarva (2022)	Spain (LaLiga)	EBIT margin	Profitability	4	5	5	91	127	0.567
Ahtiainen & Jarva (2022)	France (Ligue1)	EBIT margin	Profitability	4	5	5	115	139	−0.031
Ahtiainen & Jarva (2022)	Italy (SerieA)	EBIT margin	Profitability	4	5	5	112	137	−0.002
Ahtiainen & Jarva (2022)	England (EPL)	EBIT margin	Profitability	4	5	5	126	167	0.05
Ahtiainen & Jarva (2022)	Germany (Bundesliga)	PBT margin	Profitability	4	5	5	37	43	0.183
Ahtiainen & Jarva (2022)	Spain (LaLiga)	PBT margin	Profitability	4	5	5	91	127	0.577
Ahtiainen & Jarva (2022)	France (Ligue1)	PBT margin	Profitability	4	5	5	115	139	−0.038
Ahtiainen & Jarva (2022)	Italy (SerieA)	PBT margin	Profitability	4	5	5	112	137	−0.046
Ahtiainen & Jarva (2022)	England (EPL)	PBT margin	Profitability	4	5	5	126	167	0.074
Ahtiainen & Jarva (2022)	Germany (Bundesliga)	Leverage	Solvency	4	5	5	37	43	0.75
Ahtiainen & Jarva (2022)	Spain (LaLiga)	Leverage	Solvency	4	5	5	91	127	0.241
Ahtiainen & Jarva (2022)	France (Ligue1)	Leverage	Solvency	4	5	5	115	139	−0.254
Ahtiainen & Jarva (2022)	Italy (SerieA)	Leverage	Solvency	4	5	5	112	137	−0.198
Ahtiainen & Jarva (2022)	England (EPL)	Leverage	Solvency	4	5	5	126	167	0.039
Barajas et al. (2017)	Several countries	Quick ratio	Solvency	1	1	2	10	10	0.428
Barajas et al. (2017)	Several countries	Operating profit/Operating revenues	Profitability	1	1	2	10	10	0.724
Barajas et al. (2017)	Several countries	Equity/Total liabilities	Solvency	1	1	2	10	10	0.511
Barajas et al. (2017)	Several countries	Total operating revenues/Total assets	Profitability	1	1	2	10	10	0.559
Barajas et al. (2017)	Several countries	(Cash + short term debtors)/Current Liabilities	Solvency	1	1	2	10	10	0.302
Dimitropoulos & Koronios (2018)	Several countries	ROA	Profitability	4	5	5	388	490	0.005
Dimitropoulos & Scafarto (2021)	Italy (SerieA)	Net Profit	Profitability	5	6	6	75	90	−0.134
Dimitropoulos & Scafarto (2021)	Italy (SerieA)	Operating Profit	Profitability	5	6	6	75	90	−0.192
Dimitropoulos & Scafarto (2021)	Italy (SerieA)	Leverage = Debt/Total Assets	Solvency	5	6	6	75	90	−0.163
Fernandez-Villarino & Dominguez-Gomez (2022)	Spain (LaLiga)	Net Results	Profitability	1	1	4	44	44	0.023
Francois et al. (2022)	England (EPL)	Operating Profit/Loss	Profitability	4	6	6	77	120	0.343
Francois et al. (2022)	France (Ligue1)	Operating Profit/Loss	Profitability	6	4	6	80	118	0.034
Garcia-del-Barrio & Agnese (2023)	England (EPL)	Wages/Revenues	Profitability	1	1	9	19	20	0.466
Garcia-del-Barrio & Agnese (2023)	Spain (LaLiga)	Wages/Revenues	Profitability	1	1	9	20	20	0.526
Garcia-del-Barrio & Agnese (2023)	Italy (SerieA)	Wages/Revenues	Profitability	1	1	9	20	20	−0.716
Garcia-del-Barrio & Agnese (2023)	France (Ligue1)	Wages/Revenues	Profitability	1	1	9	20	20	−0.13
Martín-Magdalena et al. (2023)	Spain (LaLiga)	Liq Current Ratio	Solvency	4	5	8	66	81	0.068
Martín-Magdalena et al. (2023)	Spain (LaLiga)	Liq Quick Ratio	Solvency	4	5	8	66	81	0.063
Martín-Magdalena et al. (2023)	Spain (LaLiga)	Liq Cash Ratio	Solvency	4	5	8	66	81	0.621
Martín-Magdalena et al. (2023)	Spain (LaLiga)	Solvency	Solvency	4	5	8	66	81	0.752
Martín-Magdalena et al. (2023)	Spain (LaLiga)	Debt over assets	Solvency	4	5	8	66	81	0.136
Martín-Magdalena et al. (2023)	Spain (LaLiga)	EBIT Margin	Profitability	4	5	8	66	81	0.568
Martín-Magdalena et al. (2023)	Spain (LaLiga)	Net income Margin	Profitability	4	5	8	66	81	0.496
Martín-Magdalena et al. (2023)	Spain (LaLiga)	ROA	Profitability	4	5	8	66	81	0.744
Neri et al. (2021)	Italy (SerieA)	ROI	Profitability	7	7	7	137	137	−0.145
Neri et al. (2021)	Italy (SerieA)	DETEQ = D/E	Solvency	7	7	7	137	137	−0.177
Neri et al. (2021)	Italy (SerieA)	Profit	Profitability	7	7	7	137	137	0.042
Plumley et al. (2021)	England (EPL)	Z-Altman	Solvency	9	9	9	189	189	0.513
Plumley et al. (2021)	England (ELF)	Z-Altman	Solvency	9	9	9	198	198	−1.628
Plumley et al. (2021)	England (EPL)	ROA	Profitability	9	9	9	189	189	4.891
Plumley et al. (2021)	England (ELF)	ROA	Profitability	9	9	9	198	198	−0.104
Plumley et al. (2021)	England (EPL)	EBIT	Profitability	9	9	9	189	189	0.63
Plumley et al. (2021)	England (ELF)	EBIT	Profitability	9	9	9	198	198	−0.509
Urdaneta-Camacho et al. (2023)	Spain (LaLiga)	Liquidity	Solvency	1	1	3	25	25	−0.084
Urdaneta-Camacho et al. (2023)	Spain (LaLiga)	ROA	Profitability	1	1	3	25	25	0.162
Urdaneta-Camacho et al. (2023)	Spain (LaLiga)	Solvency	Solvency	1	1	3	25	25	−0.096
Urdaneta-Camacho et al. (2023)	Spain (LaLiga)	Indebtedness	Solvency	1	1	3	25	25	−0.125

**Note:** English Leagues: English Premier League (EPL) and English Football League (EFL).

**Fig. 3:**
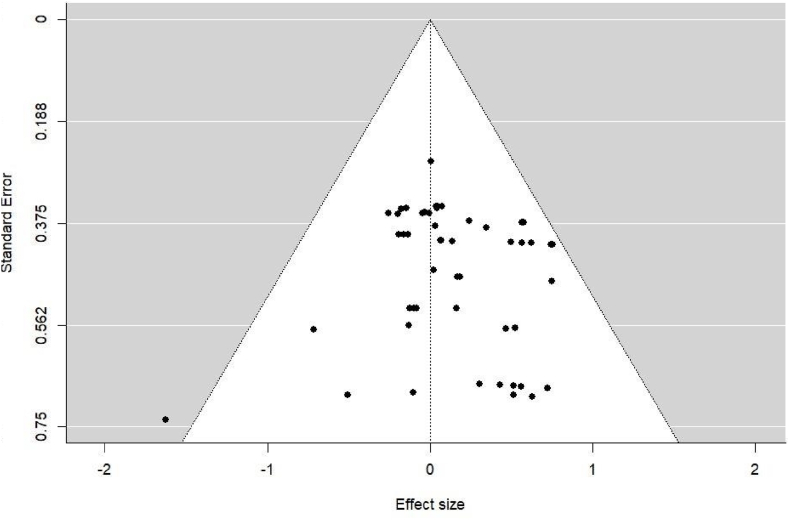
Funnel plot.

We used a funnel plot and Egger's test to analyse possible publication bias. In a funnel plot, the estimates are placed on the horizontal axis, and their standard errors (as a measure of their precision) are placed on the vertical axis. Therefore, the funnel's bottom indicates measurements with lower precision based on small samples, whereas its top indicates measurements with higher precision based on large samples. A visual inspection of the funnel plot (Fig. 3) reveals no gap in the graph on the bottom line close to zero, indicating no evidence of publication bias [66]. Owing to the funnel's slight asymmetry, which revealed more measures with positive effect sizes, we performed an Egger test to reinforce the previous conclusion regarding publication bias. This test involved conducting a linear regression between the precision of the studies (independent variable) and their effect size (dependent variable), weighted by the inverse of the variance. When no publication bias exists, the regression line originates at the origin of the Y-axis, and the further it is from zero, the greater the evidence of publication bias [67]. Therefore, a non-significant intercept of this regression is the usual method for reporting this test's result. In our case, the corresponding *p*-value was 0.505, confirming the absence of a risk of publication bias.

**Fig. 4:**
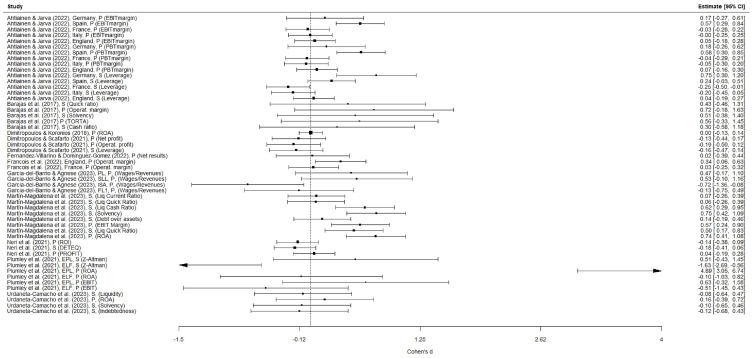
Forest plot.

As mentioned previously, the effect sizes for all measures were computed using Cohen's *d* and are graphically depicted in the forest plot (Fig. 4). This election was based on the fact that Cohen's *d* is the effect size measure most widely used and recognised in the literature [68], ensuring consistency and comparability with other studies. Additionally, although it is a specific measure to compare two conditions (as occurs in several of the studies included in our meta-analysis), the method of converting measures from correlational studies into Cohen's *d* is well established [66]. Furthermore, considering the sample sizes of the studies included in the meta-analysis, Cohen's *d* yields practically equal results to alternative effect size measures (e.g. Hedges' *g*) but is easier to interpret and compare. According to Cohen [69], depending on the *d* value, we might find a ‘small’ effect (*d* value below 0.20), a medium-sized effect (*d* value from 0.20 to 0.40), or a ‘large’ effect (*d* value higher than 0.40). The overall effect estimate was 0.109, a small effect. Indeed, it is not statistically significant (95 % CI [−0.036, 0.255], t (51) = 1.505, *p* = 0.139). This lack of statistical significance may also be attributable to the average sample size in each study (median = 146; mean = 146.13) and the high variability across studies.

As we estimated the missing standard errors for two studies ([27]; England and France), we assessed the absence of risk of bias attributable to missing results by re-running the meta-analysis without the corresponding measures. The results were highly to the previous ones (*d* = 0.101; 95 % CI [−0.062, 0.264], t (49) = 1.238, *p* = 0.221), confirming our results’ robustness.

The heterogeneity accounting for the differences in effect sizes among the studies included in the meta-analysis was large and highly significant (Q (51) = 179.742, p < 0.001). To offer a detailed breakdown of this variability's sources, we calculated the variance components at different levels. The within-studies variance component (level 2 variance) was 0.009, while the between-studies variance component (level 3 variance) was 0.084. To test whether (or not) these variance components were significantly different from zero, we performed two one-sided log-likelihood-ratio tests to compare the original model's fit (level 2 and level 3 variances freely estimated, i.e., the full model) with the fit of a restricted model wherein the variance at the level 2/3 was fixed as zero (reduced model). Significant differences in the model fit revealed the significance of variance components at the corresponding level.

The between-studies variance component was much larger than the within-studies variance component, indicating that the effect size variation was primarily attributable to differences among studies (66.32 %). By contrast, the differences between the effect sizes within the same studies were minor (7.79 %). Likelihood-ratio tests confirmed that constraining the between-studies variance to zero worsened the model fit, again indicating substantial heterogeneity in the average effect sizes between studies (Table 3). However, the results differed for the within-studies variance, which was not statistically significant.

**Table 3:** Log-likelihood-ratio test results.

	DF	AIC	BIC	LogLikehood	Likelihood-ratio test	*p*-value
Full	3	55.149	60.944	−24.575		
Reduced (level 2 variance = 0)	2	54.095	57.958	−25.047	0.945	0.165
Reduced (level 3 variance = 0)	2	70.554	74.418	17.401	17.405	<0.001

DF = degrees of freedom; AIC = Akaike information criterion; BIC= Bayesian information criterion.

The presence of heterogeneity suggested the existence of possible moderating variables. Thus, we ran corresponding three-level meta-regressions to identify possible moderators. Particularly, we tested for the moderating roles of the following variables: the *category of the measure* included in the study (profitability or solvency), whether the corresponding measure was computed using data from a *single country versus several countries*, the *number of years* analysed in the corresponding study, and the average *temporal distance* between the pre- and post-FFP measures (Table 4). Our results indicate that only the variable *category of the measure* plays a significant moderating role in explaining the variability in effect sizes. The positive estimate (β = 0.152; *p* = 0.008) indicates that the effect size is significantly larger for profitability than for solvency measures. Hence, obtaining this highly significant moderator in effect sizes is remarkable, and further description of the separate categories is warranted.

**Table 4:** Meta-regression exploration of moderating variables.

Variable	Estimate	t (50)	*p*-value
Category of measure (1 = profitability, 0 = solvency)	0.152	2.771	0.008
Single country (1 = single country, 0 = several countries)	−0.123	−0.506	0.615
Years analized	−0.001	−0.030	0.976
Temporal distance	−0.008	−0.200	0.842

Accordingly, we separately computed the effect size on the 32 profitability and 20 solvency measures. The effect size for the profitability measures was 0.151 (95 % CI [0.000, 0.302], t (31) = 2.039, *p* = 0.050), statistically significant but small according to Cohen's classification. Considering the moderate number of measures, obtaining this significant effect size is highly suggestive. However, the effect size for solvency measures was not significant, with a value of 0.049 (95 % CI [−0.166, 0.264], t (19) = 0.475, *p* = 0.639).

The positive estimate (*β* = 0.152; *p* = 0.008) indicates that the effect size is significantly different for profitability versus solvency measures—significant and positive for profitability measures (*β* = 0.151, *p* = 0.050) and nonsignificant for solvency measures (*β* = 0.049, *p* = 0.639). Therefore, this result shows that the FFP regulation's effect has been more significant for profitability than for solvency, revealing an important difference in how these financial measures respond to regulatory interventions. Therefore, these results show that the type of measure offers a novel explanation for the heterogeneity in the effect sizes of the FFP regulation on financial measures. This is a primary contribution of this study. Hence, obtaining this highly significant moderator and source of variability in effect sizes is remarkable, and further description of the separate categories is warranted. However, none of the other variables played a significant moderating role.

To offer a more descriptive view of these two categories, we performed a multigroup calculation of the effect sizes in each of the profitability and solvency groups per the proposed categorical variable country (*single country versus several countries).* Table 5 presents the results.

**Table 5:** Meta-regression exploration of moderating variables.

		Profitability					Solvency				
		K	*d*	*p*	95 % CI LB	95 % CI UB	K	*d*	*p*	95 % CI LB	95 % CI UB
**Single Country**	**No**	3	0.243	0.5119	−1.081	1.568	3	0.413	0.2544	−0.710	1.560
	**Yes**	29	0.145	0.0810	−0.019	0.311	17	0.019	0.8618	−0.212	0.251
	Spain	8	0.434	0.007	0.158	0.711	9	0.183	0.223	−0.137	0.503
	Italy	7	−0.085	0.160	−0.216	0.045	3	−0.181	0.142	−0.511	0.148
	UK	8	0.534	0.294	−0.580	1.648	3	−0.308	0.659	−2.893	2.776
	Germany	2	0.174	0.471	−1.844	2.193	1	0.750			
	France	4	−0.022	0.785	−0.257	0.213	1	−0.254			

In the profitability group, 29 (of the 32) measures correspond to only one country, with an effect size of 0.145—marginally significant. However, despite a larger effect size (0.243), the three measures from several countries were not significant. Nevertheless, this result was expected because of the low number of measurements in this subgroup. Moreover, differences were observed between the countries in terms of the number of measures and effect sizes. Hence, eight measures were from Spain, seven from Italy, eight from the UK, two from Germany, and four from France. Regarding the effect sizes, we highlighted the UK, which exhibited an effect size of 0.534, despite being non-significant, mainly because of the high variability across studies, reflected in the width of the corresponding confidence interval. Noteworthily, Spain exhibited an effect size of 0.434—highly significant, as in this case, the variability is smaller. However, France and Italy exhibited negative (though very small and, hence, non-significant) effect sizes.

In the solvency group, 17 (of the 20) measures corresponded to only one country, whose effect size is very close to zero and non-significant, as expected from the previous results. Of these, despite some being positive and others being negative, the differences were not large. The only remarkable figure corresponded to Germany, exhibiting a very high value. However, as only one such figure was noted, an inference cannot be made. The three measures corresponding to several countries yielded a higher effect size of 0.413, despite being non-significant owing to the small number of measures in the analysis.

## 4 Discussion

This study conducted a thorough review and meta-analysis of the existing literature to assess the impact of the UEFA's financial regulation on improving the European clubs' financial stability. This study aimed to address the lack of consensus in the literature and wide variation among studies. The evaluation involved a comprehensive analysis of empirical studies focusing on identifying sources of variability to measure FFP's effects on European football clubs' financial performance. Furthermore, this study aimed to determine whether the new FSR might be more effective than the FFP in enhancing European football clubs' financial performance.

This systematic review's results revealed variability in the results obtained from the studies analysed, which suggest mixed and limited effects of FFP on clubs' financial performance. This reflects the influence of contextual factors, such as national regulations and structural differences between leagues, club size and ownership structures, underlining the need for a more adaptive and specific approach to clubs' financial control policies.

The meta-analysis results revealed an important insight: the type of financial measure employed (profitability versus solvency) is a notable source of variability among studies, as its moderating effect is significant. Consequently, the FFP regulation exerted contrasting effects on club profitability and solvency. The impact on profitability has been positive and significant, whereas the effect on solvency has been non-significant, indicating that the FFP regulation has promoted short-term financial management rather than comprehensive management that supports football clubs’ long-term economic sustainability.

Furthermore, the meta-analysis results did not identify significant differences in the FFP's impact on the other moderating variables analysed, specifically exhibiting no significant differences depending on one or more countries, the number of years analysed, and the time elapsed from the period before the FFP's introduction to the last year after its implementation. Noteworthily, the impact of club size as a moderating variable could not be examined because only one study's regression model included it as such.

Therefore, our findings indicate that the FFP regulations significantly improved club's profitability but not their solvency, though some studies found that clubs have decreased their outstanding debts after the FFP's implementation [14]. We believe that a plausible reason exists to understand this different effect of the FFP based on the following study's results, but this must be studied more profoundly in the future for validation. Dimitropoulos and Scafarto [52] found a positive and significant effect between profits from the sale of player transfer and Italian clubs' financial performance following the FFP's introduction, suggesting that the FFP's introduction encouraged clubs to increase the sale of player rights to obtain greater results that would enable them to comply with the break-even rule. This can motivate clubs to improve efficiency in the sale of players and not as much in a real improvement in their resources' economic management.

Elucidating on this reasoning, we found that previous literature has demonstrated a positive relationship between profitability and solvency. When a company improves its profitability, it positively influences its solvency by increasing its financial resources, reducing debt, and increasing equity, thereby contributing to its stability and economic sustainability [70,71]. Based on this relationship between profitability and solvency, our results suggest that the positive effect on profitability has not been sufficiently consistent to improve solvency. Accordingly, this effect is plausible but should be examined in the future, and our results suggest that clubs have not yet sufficiently improved their economic efficiency after introducing the FFP.

The FFP was the first economic control regulation to impose hard budget restrictions on European clubs [23], which had previously focused solely on achieving sporting success at the expense of their economic performance, marking a significant shift [51]. These clubs operated under soft budget constraints, frequently requiring financial rescues, which was an unsustainable economic model in the long term [14]. Our results indicate that while the FFP's implementation was a first step in enhancing club profitability, we believe that it is not yet sufficient to ensure the solvency and economic sustainability of European football, contrary to studies such as that by Calahorro-López and Ratkai [25].

Available empirical evidence has suggested the need for regulatory changes to improve European football's economic control efficiency. Our results reveal the need for regulatory changes that result in football clubs focusing their financial management on not only immediate profitability but also improving both their resource management and financial structures to significantly increase their profitability and achieve solvency levels that ensure clubs' long-term financial sustainability. In this regard, the UEFA has been progressively adjusting its rules based on criticisms and recommendations from previous academic studies [23,55,57,60]. In 2022, it launched the new FSR, which maintains the previous FFP rules but includes the novelty of the cost control rule or salary cap over incomes. The new cost control rule limits spending on players based on their income, forcing club managers to manage their resources more efficiently.

Our findings indicate that this new cost control rule can increase profitability and solvency more effectively than previous regulations. This rule forces clubs to operate within their financial capabilities, which can foster greater efficiency in resource use and reduce operating costs, thereby improving profit margins and profitability. Furthermore, by controlling expenses, clubs do not need to resort to financing their operations through loans or debt, which could compromise their ability to fulfil long-term obligations, thus improving their solvency. Therefore, by limiting debt accumulation and maintaining a greater balance between income and expenses, this rule can strengthen clubs’ long-term financial sustainability.

However, as previously explained, because several clubs managed to comply nominally with the break-even rule by selling players, the UEFA has acknowledged this rule's failure to improve clubs' profitability, modifying this requirement and increasing the limit allowed for losses under certain conditions. We hold that the break-even rule and new cost control rule should be tightened and improved by limiting the impact of players' sales in their calculations, thus reinforcing the goal of improving economic efficiency and financial sustainability.

Finally, the persistence of the non-overdue debt rule, though positive, does not seem sufficient to guarantee long-term financial solvency, as explained earlier in light of our results. We posit that strengthening the regulation on debt and solvency by establishing minimum net worth to debt ratios or maximum debt to operating results ratios is necessary, which we believe would be more effective in achieving the desired objectives.

Thus, although the FFP represented a first step towards greater financial discipline, its limitations indicate that the new FSR—with a more rigorous focus on cost control—could be more effective in ensuring clubs' long-term economic stability. The new measures seem to directly address some of the shortcomings noted under FFP, such as the lack of impact on solvency, suggesting a more comprehensive and rigid approach that could improve clubs’ long-term financial sustainability.

### 4.1 Limitations

Our study had some limitations, predominantly owing to the heterogeneity and different characteristics of the studies included in our analysis. First, most studies [14,27,56] used descriptive/comparative or correlational analyses, which are not effective methods for inferring causality between FFP and financial variables. The effect of FFP is challenging, if not impossible, to isolate, as it operates jointly with numerous other variables that may impact financial measures (e.g. variables linked to the general economic environment, country-specific economic situation and/or regulation variations, particular financial reporting practices, accounting standards and transparency, management/ownership changes, investments, revenues, and team sports performance, FFP implementation timeline, or the clubs’ historical financial trends).

This fact may hide the interactions between the FFP regulation and these variables that the studies included in the meta-analysis could not capture. In this sense, only some studies [9,52,61], based primarily on regression model analysis, have incorporated some of these variables —such as club size, salary expenditure, or total assets—that affect financial performance. However, none of them have considered other possible confounding variables that could have affected both the FFP and its effect on financial performance, such as the national regulations implemented for economic control. which could have been in force in parallel with the UEFA FFP (e.g. national regulations of the English, Spanish, or French leagues) or on the distribution of television rights. To mitigate the possible existence of these confounding variables that could affect our results, we analysed the moderating role of variables such as *country*, the *years analysed*, the *temporal distance* from the initial period examined, and the final period after the FFP's implementation, which may serve as proxies for certain possible confounding variables. As previously mentioned, their moderating roles were not significant.

The possible moderating effect of club size was not incorporated in our study, because only one study considered it in its regression model. Finally, noteworthily, most measures (61.54 %) analysed in the meta-analysis corresponded to profitability measures, and only few corresponded to solvency measures (38.46 %). This implies that, until now, a greater focus has been placed on profitability; however, the FFP's objective extends beyond profitability as it seeks to improve the clubs' financial management and their sustainability and economic viability, for which a greater analysis of the effect it may have on the clubs' solvency may be needed.

### 4.2 Future research agenda

Based on the gap addressed by our study and the results obtained, we suggest an agenda for future research that advances along the following paths: First, considering that we did not find significant differences between single and multiple countries, we suggest new research focusing on a broader coverage of different leagues and local regulations' effects on clubs’ financial performance. This could include studying national economic control regulations or the distribution and negotiation of TV rights, which have not been sufficiently explored.

Second, as the effectiveness of financial regulations, such as FFP and FSR, can only be fully understood over time, it is essential to monitor clubs’ financial results and conduct longitudinal studies over several seasons. Thus, we suggest examining the impact of the FFP in the entire period wherein it has been in force; in this manner, it will be possible to examine if, over the years since its implementation and as the distance from the pre-FFP period increases, more significant effects occur than those observed in previous studies. Third, we suggest expanding the research by considering club size as a possible moderator of the FFP effect. This would allow adjustments to regulations that increase their impact on clubs, depending on their size. Fourth, considering the low number of solvency measures analysed compared to profitability measures, we suggest advancing studies that include representative means of both financial performance variables to better understand the actual effects of different rules and regulations.

Finally, considering that the FFP regulation was the first to introduce hard financial restrictions on European clubs' economic control, we suggest advancing the investigation of the effect of the degree of hard budget restrictions on a clubs' financial performance, which would allow new FSR restrictions to be evaluated and could open new possibilities for economic control to ensure European football's financial sustainability.

## 5 Conclusion

In conclusion, our study conducted a systematic review and meta-analysis to assess the FFP regulation's impact on European football clubs' financial performance and identify sources of variability across studies. We found that the type of financial measure employed is one such source. While the FFP significantly improved in profitability, it did not significantly impact solvency. The introduction of the new FSR, which includes more stringent cost control measures, aims to address these limitations and provide better financial sustainability support. Considering our findings, we believe that the new stricter economic cost control will be more effective in improving clubs' financial sustainability. However, we suggest several initiatives for improving their effectiveness.

Likewise, we hold that considering the scarce and heterogeneous studies conducted thus far, future research should not only focus on comprehensive analyses of various European leagues but also consider the entire period of FFP application; the moderating effect of club size; a balanced study of profitability and solvency measures; and the influence of other variables, such as national economic and legal regulations. These comprehensive analyses must be conducted to gain a deeper understanding of the effectiveness of both the FFP and new FSR. Such knowledge could help the European football industry's different stakeholders implement the most appropriate rules to achieve the objective of making European football financially sustainable.

## Patents and intellectual property

There are no patents to disclose.

## CRediT authorship contribution statement

**Jorge Martín-Magdalena:** Writing – review & editing, Writing – original draft, Investigation, Formal analysis, Data curation, Conceptualization. **Susana De los Ríos-Sastre:** Writing – review & editing, Writing – original draft, Investigation, Formal analysis, Data curation. **Raquel Redondo:** Writing – review & editing, Methodology, Investigation, Formal analysis. **David Alaminos:** Formal analysis, Data curation.

## Ethics statement

This manuscript represents original work and has not been published elsewhere. All authors have significantly contributed to the research and preparation of this manuscript and have approved the final version for submission. Any similarities to existing literature have been appropriately referenced and cited.

## Registration and protocol

The review was not registered, and a protocol was not prepared.

## Research support

This research received no external financial or non-financial support.

## Relationships

There are no additional relationships to disclose.

## Other activities

There are no additional activities to disclose.

## Data availability statement

As this is a study with a systematic review and a meta-analysis, the data used correspond to the articles and studies analysed, so depositing them in a publicly available repository has not been necessary. However, data will be made available on request.

## Declaration of competing interest

The authors declare that they have no known competing financial interests or personal relationships that could have appeared to influence the work reported in this paper.

## Figures and Tables

**Table:** 

Section/topic	#	Checklist item	Reported on page #
**TITLE**			
Title	1	Identify the report as a systematic review, meta-analysis, or both.	1
**ABSTRACT**			
Structures summary	2	Provide a summary including background, objectives, research methodology, results and implications.	1
**INTRODUCTION**			
Rationale	3	Describe the rationale for the review in the context of existing knowledge.	2, 3, 4
Objectives	4	Provide an explicit statement of the objective and questions the review addresses.	1, 4
**METHODS**			
Eligibility criteria	5	Specify the inclusion and exclusion criteria for the review and how studies were grouped for the syntheses.	4, 5
Information sources	6	Specify all sources searched to identify articles.	4
Search strategy	7	Present the full search strategies for all databases including any filters and limits used.	4, 5
Selection process	8	State the process for selecting studies included in the systematic review and the meta-analysis, containing how many reviewers screened each record and each report retrieved and whether they worked independently.	5
Data collection process	9	Describe the data extraction method from articles, including how many reviewers collected data, whether they worked independently, and any processes for obtaining and confirming data from researchers.	5
Data items	10	Define all outcomes for which data were sought and the methods used to decide which results to collect.	5, 6
Study risk of bias assessment	11	Specify the methods used to assess the risk of bias in the included studies, including how the reviewers assessed each study and whether they worked independently.	4, 5
Effect measures	12	Specify for each outcome the effect measures used to present results (Cohen's *d*).	5
Synthesis methods	13	Describe the methods of handling data and combining the results of studies for each meta-analysis. Describe the method to identify the presence and extent of statistical heterogeneity and the software package used.	5, 6
Reporting bias assessment	14	Describe any methods used to assess the risk of bias due to missing results in a synthesis.	5
Certainty assessment	15	Describe any methods used to assess certainty in the body of evidence for an outcome	11
**RESULTS**			
Study selection	16a	Describe the results of the search and selection process, from the number of records identified in the search to the number of studies included in the systematic review and meta-analysis, using a flow diagram.	Fig. 2
	16b	Cite studies that meet the inclusion criteria but which were excluded, and explain why they were excluded.	5, Fig. 2
Study characteristics	17	Cite each included study and present its characteristics	Table 1
Risk of bias in studies	18	Present assessments of risk of bias for each included study	11, 13, Fig. 3
Results of individual studies	19	For all outcomes for each study: (a) summary statistics for each group and (b) an effect estimate and its precision using tables and plots	Fig. 4, Table 3, Table 4, Table 5
Results of syntheses	20a	For each synthesis, briefly summarise the characteristics and risk of bias among contributing.	13, 14, Fig. 3, Fig. 4
	20b	Present the meta-analysis results of all statistical syntheses conducted and the measures of statistical heterogeneity.	11. 13, 14, 15, Fig. 4, Table 3, Table 4, Table 5
	20c	Present results of possible causes of heterogeneity among study results	15, 16, Table 4, Table 5
Reporting biases	21	Present assessments of risk of bias due to missing results for each synthesis assessed.	14
Certainty of evidence	22	Present assessments of certainty in the body of evidence for each outcome assessed	14
**DISCUSSION**			
Discussion	23a	Provide a general interpretation of the results in the context of other evidence.	15, 16, 17
	23b	Discuss any limitations of the evidence included in the review and the review processes used.	17
	23c	Discuss the implications of the results for practice, policy, and future research**.**	15, 16
**OTHER INFORMATION**			
Support	24	Declare non-financial support for the review.	18
Competing interests	25	Declare any competing interests of the authors.	18
Availability of data, code, and other materials	26	The data used is available at the reviewers' request.	18

**Table:** 

Id	Article	Journal
1	Acero et al. (2017)	Corporate Governance
2	Ahtiainen & Jarva (2022)	European Sport Management Quarterly
3	Barajas et al. (2017)	Journal of Sports Economics & Management
4	Dermit-Richard et al. (2017)	Soccer & Society
5	Dimitropoulos & Koronios (2018)	International Journal of Financial Studies
6	Dimitropoulos & Scafarto (2021)	European Sport Management Quarterly
7	Fernandez-Villarino & Dominguez-Gomez (2022)	Sport Business and Management-An International Journal
8	Franck (2018)	International Journal of Financial Studies
9	Francois et al. (2022)	Sport Business and Management-An International Journal
10	García-del-Barrio & Agnese (2023)	Managerial and Decision Economics
11	Ghio et al. (2019)	Applied Economics
12	Jakar & Gerretsen (2021)	Journal Of Sport Management
13	Maclean et al. (2022)	Qualitative Research in Financial Markets
14	Martín-Magdalena et al. (2023)	Sport Business and Management-An International Journal
15	Neri et al. (2021)	European Sport Management Quarterly
16	Nicoliello & Zampatti (2016)	Sport Business and Management-An International Journal
17	Özaydin (2020)	Russian Journal of Economics
18	Peeters & Szymanski (2012)	University of Antwerp Research Paper
19	Peeters & Szymanski (2014)	Economic Policy
20	Plumley et al. (2021)	Journal of Applied Accounting Research
21	Urdaneta et al. (2021)	Sustainability
22	Urdaneta et al. (2023)	International Journal of Financial Studies
